# Brazilian version of the Vestibular Disorders Activities of Daily Living Scale (VADL)

**DOI:** 10.5935/1808-8694.20130036

**Published:** 2015-11-02

**Authors:** Mayra Cristina Aratani, Natalia Aquaroni Ricci, Heloisa Helena Caovilla, Fernando Freitas Ganança

**Affiliations:** a PhD Student, Otorhinolaryngology and Head and Neck Surgery, Federal University of São Paulo (Physical Therapist); b Associate Professor in the Otology and Otoneurology Course in the Department of Otorhinolaryngology and Head and Neck Surgery of the Federal University of São Paulo(Associate Professor); c Adjunct Professor in the Otology and Otoneurology Course in the Department of Otorhinolaryngology and Head and Neck Surgery of the Federal University of São Paulo (Head of the Otoneurology Ward and the Vestibular Rehabilitation Program of the Otology and Otoneurology Course at the Federal University of São Paulo). Federal University of São Paulo

**Keywords:** geriatric assessment, health evaluation, translating, vestibular diseases

## Abstract

**Abstract:**

The Vestibular Disorders Activities of Daily Living Scale (VADL) assesses the impact of dizziness and body imbalance on the everyday activities of patients with vestibulopathy. The scale encompasses 28 activities divided into three sub-scales (functional, ambulation and instrumental).

**Objective:**

To translate and cross-culturally adapt the VADL to the Brazilian Portuguese language and verify its reliability.

**Method:**

Questionnaire translation methodological research. Eighty elderly subjects (age ≥ 65 years) with chronic dizziness arising from vestibular disorders were enrolled, of which 40 participated in the pre-testing stage and 40 in reliability analysis. Concordance Correlation Coefficient (CCC) analysis was used to assess reliability. Internal consistency was estimated using Cronbach's alpha (α).

**Results:**

Pre-test analysis revealed 15% of incomprehension on two activities; these items had to be adapted. The VADL-Brazil had similar levels of test-retest and inter-rater reliability for total score and presented substantial agreement (CCC = 0.79). Internal consistency was excellent for total score (α= 0.92), good for the functional (α= 0.89) and locomotion (α= 0.86) sub-scales, and poor for the instrumental subscale (α= 0.56).

**Conclusion:**

The Brazilian version of the VADL was proven adequate, with good levels of reliability and internal consistency. It might be thus considered as an alternative to assess the functional capacity of vestibulopathy patients.

## INTRODUCTION

Symptoms such as dizziness, vertigo, and body imbalance stem from vestibular disorders^1^, ^2^. These symptoms become more frequent with aging and are correlated to declining functional capacity^3^. The vestibular system undergoes a series of structural and functional modifications with aging^1^, without necessarily preventing healthy elderly individuals from effectively performing activities of daily living (ADL). However, the presence of vestibular disorders in this population introduces barriers to the realization of the ADL, consequently requiring the use of functional reserves for such activities to be performed adequately^4^. If these reserves are not enough or there is flawed postural control, elderly subjects may face situations of disability, falls, and other injuries.

A study looked into 235 elderly subjects with chronic vestibular disease and their level of performance in 15 activities assessed in the *Brazilian OARS Multidimensional Functional Assessment Questionnaire* (BOMFAQ), and found that 42% of the patients had difficulties performing seven or more activities^5^. A population study enrolled 327 community elderly individuals, assessed them using the BOMFAQ, and verified that 26.3% of the subjects faced severe impairments while performing ADL (values ≥ 7 activities)^6^. This comparison reveals the increased dependency on others and the functional incapacity the elderly with vestibular disorders may encounter. Even though the BOMFAQ was developed to study the elderly, it does not contemplate specific activities to assess dizziness or body imbalance. Other measures used frequently to assess the functional capacity of the elderly and patients in rehabilitation include the Functional Independence Measure (FIM), the Barthel index and others. Nonetheless, given their generic nature, these scales are not suitable for individuals with vestibular disease, as they fail to detect the subtle afflictions these patients face^7^.

A literature review found ten questionnaires developed specifically to assess patients with dizziness or body imbalance^8^. The most commonly used, both in research and clinical practice, are the Dizziness Handicap Inventory (DHI), the Activities-specific Balance Confidence (ABC) scale, and the Vestibular Disorders Activities of Daily Living Scale (VADL)^1^, ^8^.

The DHI and the ABC scale do not cover activities of self-care or patient mobility in detail; thus, the VADL was developed to remedy the shortcomings of these scales^4^, ^7^. The VADL stresses the assessment of activities of daily living negatively impacted by vestibular disease. The DHI is the only of these instruments to have a Brazilian Portuguese version^9^; the ABC scale is being translated into Brazilian Portuguese.

The VADL is used internationally and serves as a good alternative to assess the functional capacity of elderly subjects with vestibular disease. Therefore, this study aims to translate and cross-culturally adapt the VADL to the Brazilian Portuguese language and verify its psychometric properties.

## METHOD

This is a methodological research to translate, validate, and verify the reliability of the questionnaire. The translation and transcultural adaptation of the VADL into Brazilian Portuguese was carried out with the authorization of the author of the VADL scale in its original version in English. This study was approved by the Research Ethics Committee of the institution # 1925/09.

### The VADL instrument

The VADL scale was developed by Cohen & Kimball^2^ to assess the impact of dizziness and body imbalance on the performance of activities of daily living among patients with vestibular impairment. The VADL contemplates 28 activities divided into three sub-scales: functional (12 activities), ambulation (nine activities), and instrumental (seven activities). Each activity is assessed using a qualitative scale (0-10 points) based on the patients' self-perceived level of performance and independence while performing the activities today versus when they were free from vestibular disease^2^. The total VADL score and the scores on each sub-scale are calculated by the median score; the higher the score, the greater the patients' level of dependence and disability. Activities deemed “not applicable” (NA) by the patients are assigned a score of zero. This method prevents extreme or absent scores from interfering with the total score^4^.

### Translating the VADL

The translation of the VADL from English into Brazilian Portuguese was carried out in accordance with the recommendations proposed by the Process of Cross-Cultural Adaptation guideline^10^.

Initially, two Brazilian translators proficient in the English language translated the original instrument from English into Brazilian Portuguese. In order to enhance the clinical equivalence of the scale, one of the translators had extensive experience on otoneurology. The other translator had no previous knowledge of the concepts assessed by the scale, thus ensuring the translated version would feature language used by the population in general. The translations were analyzed in a meeting between translators and authors and, after consensus was reached, the VADL-Brazil version one was approved. The VADL-Brazil version one was then translated back into English by translators whose mother tongue was English, who had no previous knowledge on the instrument. In order to verify the transcultural equivalence of the instrument, the VADL-Brazil version one and the version translated back into English were revised by a panel of experts (a professor of methodology, a health care worker, a Portuguese professor, and the translators). The panel then approved the VADL-Brazil version two by consensus. The VADL-Brazil version two was applied as a pre-test to 40 elderly subjects with vestibular disease to verify the understanding of the scale. The difficulty understanding the language and the tasks that are not performed routinely by the Brazilian population were analyzed for each translated item. For adequacy testing purposes, items with an incomprehension rate above 15% had their content altered^11^. The distribution of answers was examined for significant numbers of omitted items or occurrence of equal responses by all respondents.

**Figure 1:**
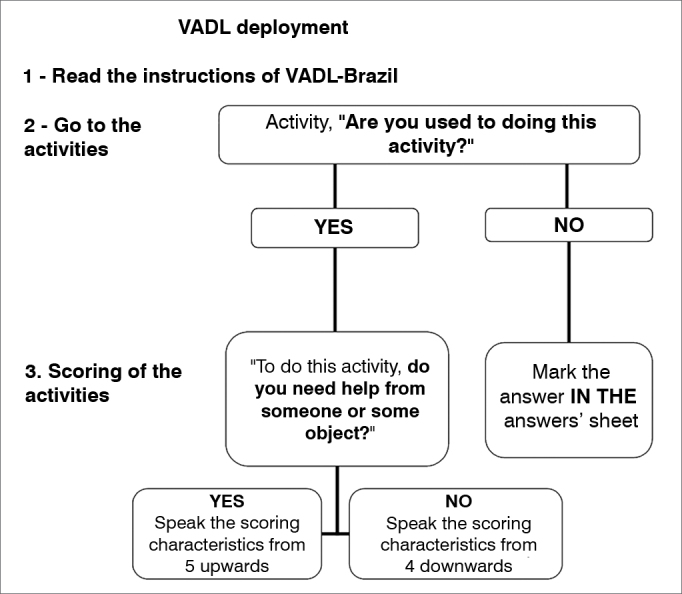
Script for the application of the Vestibular Disorders Activities of Daily Living Scale Brazilian version.

The final version of the VADL-Brazil was approved after the reports written at the end of each stage of the translation were analyzed, and the modifications indicated during pre-testing considered. The authors of the Brazilian Portuguese version of the VADL also developed a flowchart to facilitate the application of the scale through an interview (Figure 1). Though the VADL is originally self-applied, the reading difficulties seen in the sampled population due to eye disorders or low levels of education led to the adoption of an interview format instead. The VADL-Brazil interview takes about 15 minutes.

### Reliability

Inter-rater reliability was tested by the application of two independent VADL interviews by physical therapists on the same day one hour apart from each other. Test-retest reliability was verified one week later. The patients came to the health care center and were interviewed by one of the physical therapists. The interviews were carried out one week apart from each other to mitigate the risk of possible changes to the performance of the activities listed on the VADL. Both raters followed a set of standard instructions defined by the authors of the original version^2^ of the VADL and the flowchart developed for the Brazilian version while interviewing the subjects.

### Participants

The sample was made up of elderly subjects with complaints of chronic dizziness due to vestibular disease picked from the vestibular rehabilitation waiting list of an otoneurology clinic. Chronic dizziness was defined as failure to compensate for the symptom for two or more months after the triggering event^12^. Enrolled patients met the following criteria: age of 65 or older, and clinical diagnosis of chronic dizziness due to vestibular disease. Subjects with dizziness stemming from causes other than vestibular disorders, verified cognitive impairment based on the reference values for education level of the mini-mental state exam (MMSE)^13^, submitted to body imbalance rehabilitation within the last six months, or taking medication that acts on the vestibular system were excluded.

Eighty individuals were enrolled, 40 of which participated on the pre-testing stage and 40 on the reliability and psychometric analysis of the VADL-Brazil. The eligible subjects were informed of the purposes and procedures pertaining to the study, and those who decided to participate signed an informed consent term.

### Statistical analysis

Descriptive analysis was carried out to characterize the pre-test and reliability analysis sample. Floor and ceiling effects were analyzed by the presence of 15% or more of the subjects scoring one or ten on the total VADL score. Inter-rater and test-retest reliability were verified for the total score on the VADL-Brazil and its sub-scales (functional, instrumental, and ambulation). The reliability tests used with the original version in English were also carried out for the Brazilian version. Lin's concordance correlation coefficient (CCC) and Bland-Altman's method were used to that end. For the CCC, reliability was rated as poor (0.21-0.40), moderate (0.41-0.60), substantial (0.61-0.80) or high/nearly perfect (0.81-1.00)^14^. Cronbach's alpha was used to verify the internal consistency of the VADL-Brazil scale, as per the following ratings: excellent (≥0.90); good (0.90 > α≥ 0.80); acceptable (0.80> α≥ 0.70); questionable (0.70 > α≥ 0.60); poor (0.60> α≥ 0.50); and unacceptable (>0.5)^15^. The item-total correlation between the activities of the VADL-Brazil scale and the total score, as well as for its sub-scales, were analyzed through Pearson's correlation coefficient. Software packages SPSS 17.0, Stata, and Microsoft Excel were used in statistical calculations. A level of significance of 5% was adopted in all analyses.

## RESULTS

### Pre-tests

The VADL-Brazil version two was pre-tested for comprehension with 40 subjects with a mean age of 74.75 years, 30 (75%) of whom were females. Eighty percent (n = 32) of the individuals had graduated from elementary school, 15% (n = 5) from university, and 5% (n = 2) were illiterate.

Out of the 28 activities in the VADL, the subjects could not comprehend F1 (n = 5; 15%), F2 (n = 2; 5%), L13 (n = 1; 2.5%), and L14 (n = 5; 15%).

The subjects had difficulty understanding activity F1 - “sitting up from lying down” - because of the inversion on the phrase in relation to how the task is performed. Thus, the phrase was changed to “from a lying position, sit up”. Activity F2 - “standing up from sitting on the bed or chair” - presented a similar inversion, and was changed to “from a seated position, stand up” (e.g. bed or chair). After these changes were made, both activities were understood by all individuals.

L13 - “walking on level surfaces” - and L14 -“walking on uneven surfaces” were not clearly understood because of the words “on level” and “uneven”. As the illustrations “flat floor” for L13 and “with holes on it, irregular” for L14 were given, both activities were comprehended. The authors decided to keep the statements as they were with the addition of examples for L13 and L14.

Although F2 and L13 did not reach a rate of incomprehension of 15%, the authors deemed important to make changes to standardize the description of the activities in the scale.

The initial instructions were understood by all subjects. The term “vestibular disorder” in the sentence “Please choose the answer that best indicates your current level of performance on each activity against your level of performance before the onset of vestibular disorder” in the explanation of the scores was not understood by 23 individuals (57.5%). The term was replaced by “dizziness and/or body imbalance” and all subjects understood it.

All subjects comprehended the unabridged version of the score. In the abridged version, score 8 “need physical assistance” was not comprehended by four (10%) subjects and two (5%) individuals did not understand score 3 “decreased ability, no change in manner of performance”. The rate of incomprehension was below 15%, and the authors agreed that changes were not needed, as there is an explanation of the score in the unabridged version, and the abridged version is used only by the interviewer.

In terms of the activities never carried out by the individuals in the sample, answer “I have never done it” was given to activity F12 - intimate activity (eg, sexual activity) (n = 2; 5%), L21 - using an escalator (n = 1; 2.5%), I22 - driving a car (n = 20; 50%), and I26 - active recreation (n = 4; 10%). Only female subjects reported never having done these activities. Although I22 had a number high enough of responses to be removed from the scale, the authors decided to keep it, as this is one of the tasks most affected by dizziness. This finding reflects the current status of elderly Brazilian women, which is no longer the case for women from younger generations, for whom driving is a common task.

The VADL-Brazil final version is shown on Annex 1.

**Annex 1:**
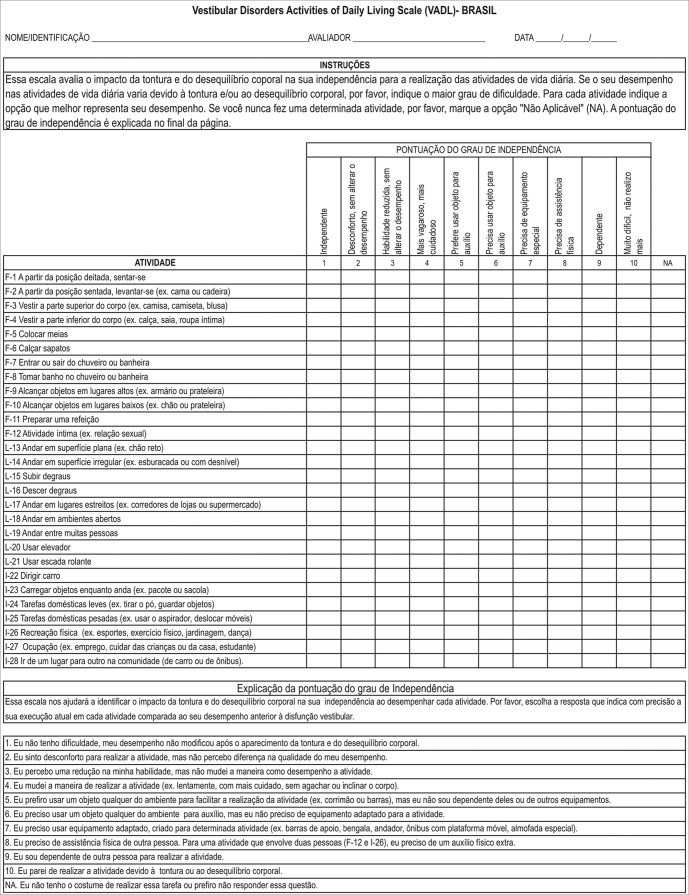
Brazilian version of the Vestibular Disorders Activities of Daily Living Scale (VADL-Brazil).

### Reliability and internal consistency of the VADL-Brazil

Forty individuals were used in reliability and internal consistency analysis. Most of the subjects were females (n = 29; 70.7%), had a mean age of 74.05 ± 6.9 years, and 5.00 ± 2.00 diseases associated to a diagnosis of vestibular syndrome. In terms of education, 77.5% of the subjects completed elementary school (n = 31), 12.5% ended high school (n = 5), 5% had a university degree (n = 2), and 5% were illiterate (n = 2). The individuals reported involvement by dizziness for 68.23 ± 76.6 months. Topographic diagnosis indicated that 70.7% had peripheral vestibular syndrome (n = 29), 20% had mixed vestibular lesions (n = 8), and 7.5% had central vestibular disorders (n = 3).

Ceiling and floor effects were not verified, as none of the subjects had a total score of 10 and 12.5% (n = 5) had a score of one.

The test-retest concordance correlation coefficients ranged from substantial - 0.75 for the functional sub-scale - to high - 0.83 in the instrumental sub-scale. Inter-rater reliability verification showed substantial concordance correlation coefficients - 0.72 in the functional sub-scale and 0.79 for total score. Bland-Altman analysis revealed that the mean test-retest and inter-rater difference was small and that no statistically significant differences were seen between the ratings assigned to the VADL and its sub-scales.

Table 1 shows the data on test-retest and inter-rater reliability, ratings 1 and 2 from rater A, and rater B's rating.

**Table 1:** Test-retest and inter-rater reliability for total score and sub-scales of the VADL-Brazil.

VADL	Rater A Measure 1	Rater A Measure 2	Rater B	Lin's CCC (CI 95%)		Bland-Altman (MD) (CI 95%)
Total score	4.06 ± 2.25	3.69 ± 3.27	4.05 ± 2.14	T-R	0.79 (0.62-0.88)	0.375 (-1.819-2.569)
				IR	0.79 (0.64-0.88)	-0.013 (-1.902-1.877)
Functional	3.86 ± 3.20	3.69 ± 3.42	3.71 ± 2.55	T-R	0.75 (0.57-0.86)	0.175 (-2.351-2.701)
				IR	0.72 (0.53-0.84)	-0.150 (-2.663-2.363)
Ambulation	4.65 ± 2.95	4.54 ± 1.94	4.55 ± 2.46	T-R	0.79 (0.60-0.89)	0,113 (-1.939-2.164)
				IR	0.78 (0.62-0.88)	-0.100 (-1.902-1.877)
Instrumental	3.85 ± 5.93	3.84 ± 5.22	4.13 ± 6.21	T-R	0.83 (0.70-0.91)	0.013 (-2.728-2.753)
				IR	0.78 (0.62-0.88)	0.275 (-2.948-3.498)

MD: Mean difference; T-R: Test-retest; IR: Inter-rater.

In terms of the VADL internal consistency, the VADL total score had an excellent rating (α= 0.92), the functional (α= 0.89) and ambulation (α= 0.86) sub-scales had a good rating, and the instrumental sub-scale had a poor rating (α= 0.56).

Tables 2 and 3 show the item-total correlation values and Cronbach's alpha for total score and individual sub-scale scores on VADL-Brazil.

**Table 2:** Internal consistency of items in the VADL-Brazil scale with total score.

Item	Item-total correlation	Cronbach's alpha
F1 sitting up from lying down	0.66	0.92
F2 standing up from sitting on the bed or chair	0.62	0.92
F3 dressing the upper body	0.66	0.92
F4 dressing the lower body	0.58	0.92
F5 putting on socks or stockings	0.52	0,93
F6 putting on shoes	0.46	0,93
F7 moving in or out of the bathtub or shower	0.70	0.92
F8 bathing yourself in the bathtub or shower	0.67	0.92
F9 reaching overhead	0.55	0.92
F10 reaching down	0.67	0.92
F11 meal preparation	0.71	0.92
F12 intimate activity	0.34	0,93
A13 walking on level surfaces	0.51	0,93
A14 walking on uneven surfaces	0.63	0.92
A15 going up steps	0.61	0.92
A-16 going down steps	0.65	0.92
A-17 walking in narrow spaces	0.66	0.92
A-18 walking in open spaces	0.69	0.92
A-19 walking in crowds	0.64	0.92
A-20 using an elevator	0.34	0,93
A-21 using an escalator	0.56	0.92
I-22 driving a car	-0.27	0,94
I-23 carrying things while walking	0.64	0.92
I-24 light household chores	0.61	0.92
I-25 heavy household chores	0.28	0,93
I-26 active recreation	0.01	0,93
I-27 occupational role	0.72	0.92
I-28 traveling around the community	0.73	0.92
Total	-	0.92

**Table 3:** Internal consistency of items in the VADL-Brazil scale with individual scores in each sub-scale.

Item	Item-total correlation	Cronbach's alpha
F1	0.74	0.87
F2	0.64	0.88
F3	0.68	0.88
F4	0.57	0.88
F5	0.55	0.89
F6	0.49	0,89
F7	0.69	0.88
F8	0.61	0.88
F9	0.63	0.88
F10	0.68	0.88
F11	0.70	0.88
F12	0.23	0.90
Functional	-	0.89
L13	0.51	0.86
L14	0.66	0.84
L15	0.63	0.85
L16	0.66	0.84
L17	0.58	0.85
L18	0.74	0.83
L19	0.66	0.84
L20	0.30	0.88
L21	0.61	0.85
Ambulation	-	0.86
I22	-0.25	0.70
I23	0.58	0.40
I24	0.44	0.46
I25	0.22	0.55
I26	0.09	0.59
I27	0.68	0.35
I28	0.40	0.48
Instrumental	-	0.56

The activities that better represented the results of the VADL scale in the item-total correlation for total score were F7 (moving in or out of the bathtub or shower), F11 (meal preparation), I27 (occupational role), and I28 (traveling around the community [car, bus]). In the functional sub-scale the more representative activity was F1 (from the lying position, sit up). In the ambulation sub-scale, activity L18 (walking in open spaces) was the more representative, while activity I27 (occupational role) was the more representative for the instrumental sub-scale.

Activity I22 (driving a car) had the lowest correlation values for total score and the instrumental sub-scale.

## DISCUSSION

In this study, the elderly sample, were most composed by females with elementary school education. The individuals enrolled in the study designed to develop the VADL scale in the United States^2^ were adults and their level of education was not reported. Additionally, they answered the questionnaire by themselves. Given the characteristics of the subjects in this study and supported by the application of other instruments devised to assess the functional capacity of elderly individuals, the authors chose to administer the VADL-Brazil in the form of an interview. A script was developed to standardize and facilitate the use of the instrument in Brazil.

Beaton et al.^10^ recommend that the final version of questionnaires translated into other languages be comprehended by respondents in an equivalent manner to how a 12-year-old individual would, i.e., a person with a minimum of six years of school education. The final version of the VADL-Brazil was adequate for the elderly population, as it was comprehended by subjects with a wide range of educational backgrounds (from illiterate subjects to university graduates). Despite the subjects' low overall level of education, only four of the activities in the VADL-Brazil scale were not comprehended at first and had their contents modified. In the explanation of the instrument, the technical term “vestibular disorder” was not easily understood by most of the individuals in the sample, and was replaced by the colloquial term “dizziness and/or imbalance”. The employment of everyday words and expressions makes it easier for subjects to understand what is asked of them in the scale, without losing the equivalence between languages.

During pre-testing it was found that activity I22 (driving a car) had never been carried out by half of the subjects in the sample. Therefore, this activity could have been removed from the instrument. However, the authors chose to keep it in the VADL-Brazil scale because this finding reflects the reality of this cohort tested, and not that of the population in general in whom the instrument can be applied. Cohen & Kimball^2^ had a similar problem with activity I28 (traveling around the community), which initially revolved around the use of public transportation (buses or trains). As most subjects did not use public transportation - a characteristic of that local population - the authors decided to include the possibility of using a car in this activity. But the option of riding a bus was kept, so that proper attention was paid to the importance of public transportation in other communities. An additional reason to keep activity I22 (driving a car) as part of the instrument is the fact that the VADL offers respondents the possibility of answering NA (not applicable) and give it a score of zero, thus not impacting the total score as it is calculated based on the median value of all scores.

Therefore, one may state that the VADL is a questionnaire that comprises the basic and instrumental activities of daily living despite the social or environmental context, as none of the activities in the original version had to be excluded from the Brazilian Portuguese rendering of the instrument.

It is recommended that every instrument translated and adapted into another language and culture be submitted to psychometric assessment and seen to perform similarly to its original version^10^. In terms of reliability, the VADL-Brazil produced results similar to the ones reported on the study carried out to develop the scale^2^. However, in the original version^2^ only test-retest reliability was verified, and the VADL was responded by adult patients (n = 17) twice in sessions two hours apart from each other. Inter-rater reliability was not tested, as the questionnaire was answered by the individuals. According to Duracinsky et al.^8^, in a review in instruments used to assess the impact of dizziness in patients with vestibular disease, reliability assessment was not properly done for most questionnaires, the VADL scale included. The reason is that the samples should include more than 30 people and the test-retest interval need to be longer than a week. In order to address the pointed issues^8^, in this study the VADL had test-retest reliability assessed with the instrument being applied to 40 subjects twice with an interval of one week between the first and second interviews. And to complete the analysis, inter-rater reliability was also tested.

This study showed reliability ratings from substantial to high on test-retest, whereas Cohen & Kimball^2^ saw superior results with perfect reliability for total score and all sub-scales. The difference in the results may stem from the type of sample, the way in which the VADL was applied, factors related to memory and the time elapsed between tests. Despite the differences, the VADL is a reliable instrument in the original and Brazilian versions.

The VADL features a quite extensive scoring range (one to 10 points) in functional terms, and applying it can be somewhat challenging^7^. Thus, inter-rater disagreement may occur more commonly than in scales with less detailed answers, ie. yes or no. That way to guarantee reliable answers it is important to training raters on how to apply the scale and use the scoring flowchart proposed for the VADL-Brazil.

When looking at internal consistency, Cohen & Kimball^2^ observed excellent values for total score (α= 0.97) and the functional (α= 0.92), ambulation (α= 0.96), and instrumental (α= 0.91) sub-scales. Values of such order of magnitude were also seen for total score (α= 0.92) in the VADL-Brazil. The elevated internal consistency of the total score in the VADL-Brazil scale shows that the activities measure one same construct, i.e., the functional capacity of patients suffering from dizziness.

Unlike the original study^2^, the instrumental sub-scale had poor internal consistency (α= 0.56). This result was observed because this sub-scale contained activities to which ‘not applicable' was a frequent answer, such as I22 (driving a car) and I26 (active recreation). Activity I22 had the poorer correlation and had significant impact on the internal consistency of the instrumental sub-scale. If activity I22 were not considered, Cronbach's alpha would move to 0.70 - an acceptable rating. Therefore, according to psychometric analysis, activity I22 should be removed from the instrument. However, Cohen^7^ stressed the relevance of looking into the problems individuals with dizziness encounter when driving. Patients in this situation have reported difficulty driving in circumstances of poor visibility (at night, in rain or fog), steep terrain, and environments with optokinetic stimulation (intense moving traffic)^7^. Thus, activity I22 was kept as part of the scale.

The Brazilian Portuguese version of the VADL scale was shown to be adequate and reliable when applied to elderly subjects with comorbidities and functional incapacity. The scale is expected to perform similarly when used with younger individuals. Additional studies are being carried out to strengthen the metric quality of the instrument.

## CONCLUSION

Assessing the impact of dizziness and vertigo upon the daily lives of the patients from their perspective is essential for therapy planning. However, it is important that health care workers use relevant and valid questionnaires from the literature^8^ when performing such assessment. The VADL-Brazil is a new tool to be used in our country to explore the functional capacity of individuals with vestibular diseases and guide therapy planning and vestibular rehabilitation in particular.
